# Proteomic analysis indicates that mitochondrial energy metabolism in skeletal muscle tissue is negatively correlated with feed efficiency in pigs

**DOI:** 10.1038/srep45291

**Published:** 2017-03-27

**Authors:** Liangliang Fu, Yueyuan Xu, Ye Hou, Xiaolong Qi, Lian Zhou, Huiying Liu, Yu Luan, Lu Jing, Yuanxin Miao, Shuhong Zhao, Huazhen Liu, Xinyun Li

**Affiliations:** 1 Key Laboratory of Agricultural Animal Genetics, Breeding, and Reproduction of the Ministry of Education & Key Laboratory of Swine Genetics and Breeding of Ministry of Agriculture, Huazhong Agricultural University, Wuhan, 430070, P. R. China; 2 The Cooperative Innovation Center for Sustainable Pig Production, Wuhan, 430070, P. R. China

## Abstract

Feed efficiency (FE) is a highly important economic trait in pig production. Investigating the molecular mechanisms of FE is essential for trait improvement. In this study, the skeletal muscle proteome of high-FE and low-FE pigs were investigated by the iTRAQ approach. A total of 1780 proteins were identified, among which 124 proteins were differentially expressed between the high- and low-FE pigs, with 74 up-regulated and 50 down-regulated in the high-FE pigs. Ten randomly selected differentially expressed proteins (DEPs) were validated by Western blotting and quantitative PCR (qPCR). Gene ontology (GO) analysis showed that all the 25 DEPs located in mitochondria were down-regulated in the high-FE pigs. Furthermore, the glucose-pyruvate-tricarboxylic acid (TCA)-oxidative phosphorylation energy metabolism signaling pathway was found to differ between high- and low-FE pigs. The key enzymes involved in the conversion of glucose to pyruvate were up-regulated in the high-FE pigs. Thus, our results suggested mitochondrial energy metabolism in the skeletal muscle tissue was negatively correlated with FE in pigs, and glucose utilization to generate ATP was more efficient in the skeletal muscle tissue of high-FE pigs. This study offered new targets and pathways for improvement of FE in pigs.

Feed cost currently accounts for more than 60% of the total costs of pig production. Thus, improving feed efficiency (FE) is an important approach for reducing pig production expenses. Currently, two highly correlated indicators, namely, residual feed intake (RFI) and feed conversion ratio (FCR), are being used to measure the FE trait^1^,^2^,^3^. Low levels of FCR/RFI indicate high FE^4^. FE is a complex economic trait that is known to be correlated with many biological factors. In pigs, the growth rate and skeletal muscle content have been reported to be positively related to the FE trait^5^,^6^ Further, the fat content and physical activity have been found to be negatively related to the FE trait^7^,^8^.

Previous studies have identified some quantitative trait loci (QTLs) and candidate genes of the FE trait. QTL mapping studies have shown the existence of two important QTL regions, particularly, 30.5–31.5 Mb on pig chromosome 1 (SSC1) and 120.5–121.5 Mb on SSC9 for RFI^9^. Further, three FCR QTLs, located on SSC2, SSC7, and SSC9, have been identified in genome-wide association studies (GWAS)^10^. It has also been reported that four loci containing 13 single-nucleotide polymorphisms (SNPs) located on SSC4, SSC7, SSC8, and SSC14 are related to the FE trait in pigs^11^. Several studies have reported that the MC4R gene is an important determinant of FE^12^,^13^,^14^,^15^. Moreover, XIRP2, TTC29, SOGA1, MAS1, GRK5, PROX1, GPR155, and ZFVE26 have also been recognized as candidate genes for RFI in pigs^16^.

The molecular mechanisms of the FE trait have been partially revealed by transcriptome analysis. It has been reported that genes involved in lipogenic and steroidogenic pathways are down-regulated in the liver and fat tissues of high-FE pigs^17^. Also, transcriptome analysis has shown that the signaling pathway of ESR1 could affect the FE trait by regulating acute caloric restriction of pigs^17^. Our recent study showed that the signaling pathway of vitamin A metabolism in liver tissue is related to FE in pigs^18^. Moreover, in skeletal muscle tissues, the signaling pathways of muscle growth and mitochondrial energy metabolism were found to be related to FE in pigs. The expression levels of five key energy metabolism genes, namely, FABP3, RCAN, PGC-1A, HK2, and PRKAG2, in skeletal muscle tissues were negatively related to FE in pigs^19^. Whole-blood transcriptome analysis indicated that the FE trait could also be affected by immune response, inflammatory response, and anti-apoptotic physiological processes^20^. Although many studies focus on signaling pathways and candidate genes of FE trait, only few of them have been identified, and the mechanisms underlying FE remain largely unknown.

Moreover, although some studies have investigated the FE trait at the transcription level, only a few studies have been performed at the protein level. In this study, we systematically analyzed the differentially expressed proteins (DEPs) in the skeletal muscle tissues of high- and low-FE pigs using the iTRAQ approach. First, total proteins were isolated from the skeletal muscle tissues of extremely high- and extremely low-FE pigs. Then, iTRAQ labeling technology was used to identify the DEPs between these two groups. Further, randomly chosen DEPs were validated using Western blotting and quantitative PCR (qPCR) methods. Finally, functional analysis revealed that mitochondrial energy metabolism and cytoplasm glucose metabolism are closely related to FE in pigs.

## Results

### A total of 1780 proteins identified in the skeletal muscle tissue of pigs using the iTRAQ method

**Figure 1:**
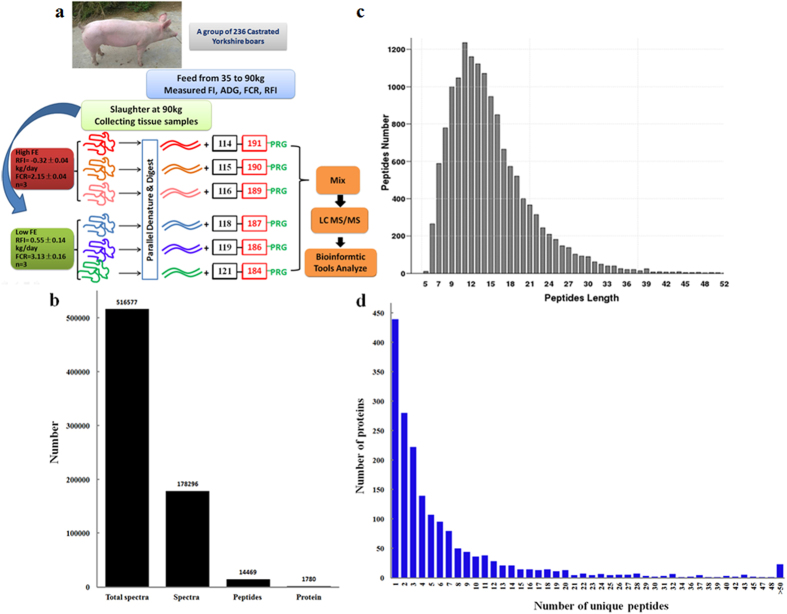
Proteomic analysis using the iTRAQ method. (**a**) Workflow for the iTRAQ method. The samples of three high-FE pigs were labeled with 114, 115, and 116 iTRAQ tags. The samples of three low-FE pigs were labeled with 118, 119, and 121 iTRAQ tags. (**b**) Total spectrum, peptides and proteins identified by the iTRAQ method. (**c**) Distribution of peptide lengths. (**d**) Distribution of proteins based on the number of peptides.

In this study, the FE trait of 236 Yorkshire pigs were first measured. The proteins of the skeletal muscle tissues of three pigs with extremely high-FE and three with extremely low-FE were extracted for further study. The proteome of the six samples was determined by iTRAQ integrated with LC–MS/MS (Fig. 1a). A total of 516577 raw spectra were generated, from which 178296 spectra, 14469 peptides, and 1780 proteins were then identified using the Uniprot Pig database (Fig. 1b). The lengths of most peptides ranged from 6 to 30 amino acids, with the maximum number of proteins being 11 amino acids long (Fig. 1c). Among the 1780 identified proteins, more than 75% contained at least two peptides (Fig. 1d).

### A total of 124 differentially expressed proteins between the high- and low-FE pigs

After identification of the proteins, the DEPs were further analyzed. In total, the expression levels of 124 proteins were significantly different between the high- and low-FE pigs (up-regulated ≥ 1.2 or down-regulated ≤ 0.84; p ≤ 0.05). Among the 124 DEPs, 74 were up-regulated and 50 were down-regulated in the high-FE pigs (Table S1). The top 10 up-regulated and down-regulated proteins in the high-FE pigs were listed in Table 1. Among the top 10 down-regulated proteins, 5 belonged to the keratin protein family, which includes KRT1, KRT5, KRT10, KRT75, and LOC100621639 (keratin, type II microfibrillar, and component 7C-like proteins).

### GO and KEGG pathways analysis of the DEPs

**Figure 2:**
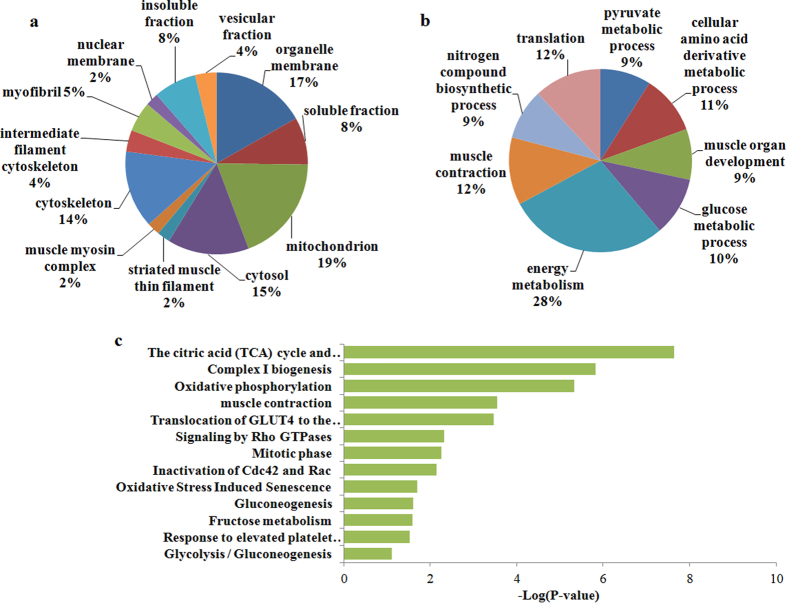
Functional analysis of the DEPs between the high- and low-FE pigs. (**a**) Cellular components based on GO analysis. (**b**) Biological processes based on GO analysis. (**c**) Most significant pathways based on the KEGG database.

The functions of DEPs were further analyzed by using the online DAVID. Results of cellular component analysis showed that 19% of the proteins were located in the mitochondria, 17% in organelle membranes, 15% in the cytosol, and 14% in the cytoskeleton (Fig. 2a). Meanwhile, results of biological process analysis indicated that 28% of the proteins were related to energy metabolism, 12% to muscle contraction, 12% to translation, 11% to cellular amino acid derivative metabolic process, and 10% to glucose metabolism (Fig. 2b). Results of KEGG pathway analysis revealed that the tricarboxylic acid (TCA) cycle and electron transport respiratory chain, complex I synthesis and oxidative phosphorylation pathways were the top three significant pathways (Fig. 2c).

### The proteins involved in mitochondrial energy metabolism and glucose metabolism were differentially expressed between the high- and low-FE pigs

**Figure 3:**
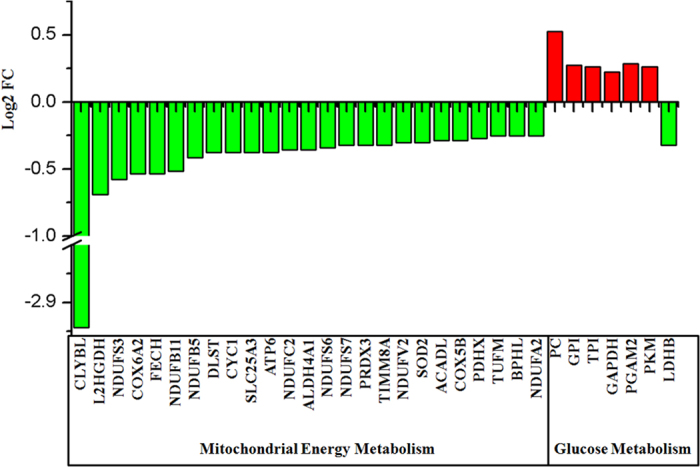
DEPs located in the mitochondria and DEPs participating in glucose metabolism. The red and green bars represent up-regulated and down-regulated proteins, respectively, in high FE-pigs compared to low-FE pigs.

Based on GO and KEGG pathway analyses, the DEPs were mainly related to mitochondrial energy metabolism and glucose metabolism. In total, 25 differentially expressed mitochondria-located proteins were down-regulated in the high-FE pigs, which included TUFM, DLST, NDUFA2, NDUFB11, NDUFB5, FECH, NDUFC2, BPHL, PRDX3, ACADL, CLYBL, COX5B, TIMM8A, SOD2, NDUFS7, NDUFS6, L2HGDH, NDUFV2, SLC25A3, COX6A2, ALDH4A1, NDUFS3, PDHX, ATP6, and CYC1. Meanwhile, seven important proteins for glucose metabolism were differentially expressed. Among these, LDHB was significantly down-regulated, while PKM, PC, GAPDH, GPI, TPI1, and PGAM2 proteins were up-regulated in the high-FE pigs (Fig. 3).

### Validation of the DEPs identified by iTRAQ

**Figure 4:**
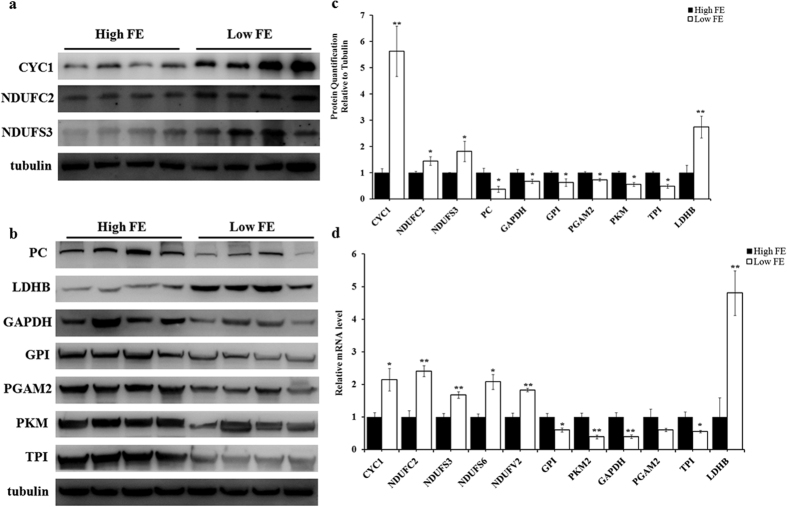
Validation of DEPs participating in glucose and mitochondrial energy metabolism. (**a**) Western blotting results of CYC1, NDUFC2, and NDUFS3 mitochondria -located proteins in the skeletal muscle tissue of high- and low-FE pigs. (**b**) Western blotting results of PC, LDHB, GPI, PKM, TPI1, PAGM2, and GAPDH proteins, which are known to participate in glucose metabolism, in the skeletal muscle tissue of high- and low- FE pigs. (**c**) Statistical analysis of Western blotting results. The results were presented as mean ± S.E.M (n = 14, 7 high-FE pigs Vs 7 low-FE pigs). The mean value of high- FE group was set as 1. *P < 0.05; **P < 0.01. The α-Tubulin protein was used as internal control. (**d**) Q-PCR results of CYC1, NDUFC2, NDUFS3, NDUFS7, NDUFV2, LDHB, PKM, TPI1, GPI, PAGM2 and GAPDH genes. The results are presented as mean ± S.E.M (n = 8, 4 high-FE pigs Vs 4 low-FE pigs). The mean value of high- FE group was set as 1. *P < 0.05; **P < 0.01. Levels of α-tubulin mRNA was used as internal control.

To confirm the DEPs identified by iTRAQ, 10 of the 32 DEPs shown in Fig. 3 were randomly selected for Western blotting. This included 3 mitochondrial-located proteins (CYC1, NDUFC2, and NDUFS3), and 7 key glucose metabolism proteins (PC, LDHB, GPI, PKM, TPI1, PAGM2, and GAPDH). Moreover, 7 high- FE pigs and 7 low- FE pigs, which included the 6 pigs from iTRAQ assay and other 8 of the 236 total pigs, were used for Western blotting detection. The results of Western blotting were exactly the same as those of iTRAQ (Fig. 4a–c). Furthermore, 11 DEPs were validated at the transcriptional level by qPCR, which included CYC1, NDUFC2, NDUFS3, NDUFS7, NDUFV2, LDHB, PKM, TPI1, GPI, PAGM2, and GAPDH. The qPCR results were similar to iTRAQ (n = 8; 4 high-FE pigs Vs 4 low-FE pigs) (Fig. 4d).

### The glucose–pyruvate–TCA–oxidative phosphorylation energy metabolism signaling pathway in the skeletal muscle tissue plays important roles in FE regulation in pigs

**Figure 5:**
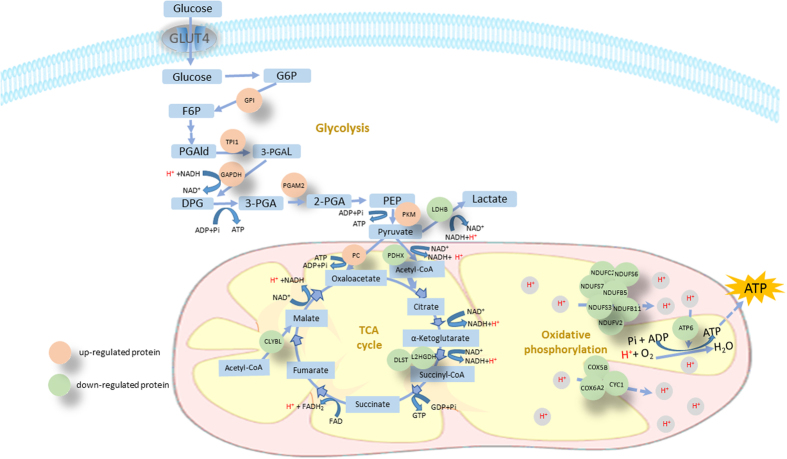
Key signaling pathways represented by DEPs in skeletal muscle tissues between high- and low-FE pigs. Pink represents up-regulated proteins and green represents down-regulated proteins in high-FE pigs.

To identify the important pathways involved in FE regulation, we subjected the DEPs to signaling pathway analysis. We found that the glucose–pyruvate–TCA–oxidative phosphorylation energy metabolism signaling pathway was the most significantly enriched pathway based on KEGG and REACTOME databases. Further, the pathway image, illustrated using the Cytoscape software, is shown in Fig. 5. In this pathway, PKM, GAPDH, GPI, TPI1, and PGAM2 proteins, which catalyze the conversion of glucose to pyruvate, were significantly up-regulated in the high-FE pigs (P < 0.01). Furthermore, the PC protein, which catalyzes the conversion of pyruvate to oxaloacetate, was significantly up-regulated in high-FE pigs (P < 0.01). On the other hand, LDHB and PDHX, which catalyzes the conversion of pyruvate to lactate and conversion of pyruvate to acetyl-coA, respectively, were down-regulated in the high-FE pigs (P < 0.01). All the 11 key proteins in the oxidative phosphorylation reaction, including NDUFS7, NDUFS6, NDUFB5, NDUFV2, NDUFC2, NDUFS3, NDUFA2, COX6A2, COX5B, ATP6, and CYC1, were significantly down-regulated in the high-FE pigs (P < 0.01) (Fig. 5). In addition, the DLST, L2HGDH, and CLYBL proteins, which catalyzed the important steps of the TCA cycle, were significantly down-regulated in the high-FE pigs (P < 0.01).

## Discussion

In this study, we have systematically analyzed the proteome of longissimus muscle in high- and low-FE pigs. The DEPs and important signaling pathways related to the FE trait have been identified. We observe that all the DEPs located at the mitochondria are significantly down-regulated in the high-FE pigs. Based on these results, we finally conclude that mitochondrial energy metabolism in the skeletal muscle is negatively correlated with FE in pigs.

More than 90% of total energy is known to be generated by the mitochondria via the TCA and oxidative phosphorylation processes^21^. In this study, 3 proteins in the TCA and 11 proteins in oxidative phosphorylation processes were found to be significantly down-regulated in high-FE pigs compared to low-FE pigs. The results indicate that ATP synthesis is comparatively lower in the skeletal muscle tissues of high-FE pigs. One previous study showed that oxidative metabolism in skeletal muscle, as well as superoxide dismutase and glutathione peroxidase 3, were down-regulated in the skeletal muscle tissue of high-FE pigs at the transcription level^22^. Our previous study also found that most mitochondria-located genes were down-regulated at the transcriptional level in the skeletal muscle tissue of high-FE pigs^19^. We deduce that mitochondrial energy metabolism in skeletal muscle tissue is related to FE in pigs. It is well known that the skeletal muscle is the largest tissue in pigs and accounts for 40–50% of the total body weight^23^. Furthermore, muscle contraction and basal metabolism consume large amounts of energy. Therefore, moderately restricted energy metabolism in skeletal muscle tissue could benefit FE in pigs.

In this study, the glucose–pyruvate–TCA–oxidative phosphorylation pathway in the skeletal muscle tissue has been identified as a key pathway in the regulation of FE trait in pigs. In this pathway, all proteins catalyzing the cytoplasmic conversion of glucose to pyruvate are up-regulated in the high-FE pigs. Interestingly, the enzyme that catalyzes the conversion of pyruvate to oxaloacetate was up-regulated, whereas the enzymes that catalyze the conversion of pyruvate to lactate and pyruvate to acetyl-CoA were down-regulated. These results indicate that the access of cytoplasmic glucose to the TCA process could be promoted through the production of mitochondrial oxaloacetate from pyruvate by the pyruvate carboxylase. Furthermore, TCA was inhibited in the high-FE pigs based on the down-regulation of the key catalytic proteins. Thus, we hypothesize that the concentration of some TCA metabolites were higher in the skeletal muscle of high than low-FE pigs, which could increase the cellular energetic charge and negatively influence the mitochondrial oxidative metabolism. A previous study also indicated that the activities of lactate dehydrogenase, citrate synthase and beta-hydroxyacyl-CoA dehydrogenase (fatty acid beta-oxidation) were reduced in the longissimus muscle of low RFI pigs^24^. Therefore, our findings are consistent with previous studies denoting a reduction in skeletal muscle oxidative metabolism in high Vs low-FE pigs^22^. Hence, our data suggest that cytoplasmic glucose could access the TCA process more directly and faster in the skeletal muscle tissue of high-FE compared to low-FE pigs.

Besides, it has been confirmed that extensive oxidative phosphorylation accompanies greater amount of heat production in less feed efficient pigs^25^. In the present study, all the enzymes involved in oxidative phosphorylation process were down-regulated in high-FE pigs. Therefore, we deduce that an equal glucose amount could generate greater ATP and less heat in the muscle tissue of high-FE pigs. Thus, we hypothesize that moderately restricting oxidative phosphorylation would improve the efficiency of ATP generation and reduce the waste of energy, thereby promoting the FE.

In conclusion, the proteome of the skeletal muscle tissues of high- and low-FE pigs have been comparatively analyzed in this study. All of the differentially expressed mitochondria-located proteins have been significantly down-regulated in the high-FE pigs. This observation reveals that mitochondrial energy metabolism in skeletal muscle tissues is negatively correlated with FE in pigs. Moreover, the glucose–pyruvate–TCA–oxidative phosphorylation pathway has been identified as one of the key pathways of FE in pigs. Also, our results suggest that the access of glucose to TCA through the conversion of pyruvate to oxaloacetate by pyruvate carboxylase was higher in the skeletal muscle of high-FE pigs. Thus, our results show that moderately restricting the oxidative energy metabolism of skeletal muscle could be beneficial for improving FE in pigs.

## Methods

### Animals and tissues

In this study, the ad libitum feed intake of 236 castrated purebred boar Yorkshire pigs were measured using ACEMA 64 automated individual feeding systems at the Agricultural Ministry Breeding Swine Quality Supervision Inspecting and Testing Center (Wuhan, P. R. C.). The experimental protocols, diet and pig performance data have been reported previously^19^. Three pigs with extremely high-FE (RFI = −0.32 ± 0.04 kg/day; FCR = 2.15 ± 0.04) and three with extremely low-FE (RFI = 0.55 ± 0.14 kg/day; FCR = 3.13 ± 0.16) were selected from the 236 pigs. The FE of these two groups were significantly different (P < 0.01)^19^. These six pigs were used for iTRAQ assay. Further, 14 pigs (7 high-FE pigs Vs 7 low-FE pigs) and 8 pigs (4 high-FE pigs Vs 4 low-FE pigs) were used for Western blotting and qPCR validation experiments, respectively. The performance of the 14 pigs was shown in Supplementary Table S2. The skeletal muscle tissues of all the pigs were first collected from the same location in longissimus muscle within 30 min after slaughter (body weight, 90 kg). Then, these samples were snap frozen in liquid nitrogen and stored at −80 °C. All procedures were carried out in accordance with the approved guidelines of Regulation of the Standing Committee of Hubei People’s Congress (Wuhan, P. R. C.). The experimental protocols were approved by the ethics committee of Huazhong Agricultural University (HZAUMU2013-0005) (Wuhan, P. R. C.).

### Protein preparation and iTRAQ labeling

For iTRAQ analysis, the six samples were ground to small pieces in liquid nitrogen and lysed in specific buffer solution (4% sodium dodecyl sulfate, 1 mM dithiothreitol, and 150 mM Tris–HCl at pH 8.0) and protease inhibitor (Roche, USA). Then, the lysed proteins were sonicated on ice. The proteins were then isolated by centrifugation at 20,000 *g* for 20 min at 4 °C. Protein concentration was measured using a Bradford Protein Assay Kit (Thermo Fischer Scientific, USA). Then, 100 μg protein of each sample was transferred to a 1.5 ml new tube and treated with 2 μg trypsin for 12 h at 37 °C. After digestion, the peptide solution was dried by vacuum centrifugation and redissolved in 0.5 M tetraethyl-ammonium bromide (TEAB). Finally, iTRAQ labeling was carried out according to the kit manufacturer’s instructions (AB Sciex UK Limited). The three samples from high-FE pigs were labeled with 114, 115, and 116 iTRAQ tags, respectively, and the three samples from low-FE pigs were labeled with 118, 119, and 121 iTRAQ tags, respectively.

### Liquid chromatography (LC)–tandem mass spectrometry (MS/MS)

The six iTRAQ labeling samples were mixed into one sample and analyzed using a Triple TOF 5600 system (AB Sciex, USA) connected to a Nanoflex microchip. The peptides of the samples were subjected to chromatography for 90 min under the following conditions: gradient concentration of 2–30% Buffer A (0.1% formic acid, 5% acetonitrile)+Buffer B (0.1% formic acid, 95% acetonitrile). The MS1 spectra was collected at 250 ms in the 350–1,500 m/z range. The top 20 more intense precursors with charge states 2–5 were selected and fragmented for the second round of mass analysis. MS2 spectra was then collected at 100 ms in the 50–2,000 m/z range.

### Bioinformatics analysis

The raw MS/MS data was converted to MGF file using the 5600 masconverter software (AB Sciex, USA). Proteins were identified using the Uniprot Pig database (http://www.uniprot.org) and the Mascot search engine (Matrix Science, London, UK; version 2.3.02). The identification parameters were set as follows: mass tolerance of 25 ppm, fragment ion mass tolerance of 0.1 Da. Variable modifications: oxidation (M), acetyl (protein N-term), Gln- > pyro-Glu (N-termQ), iTRAQ8plex (Y); fixed modifications: methylthio (C), iTRAQ8plex (N-term), and iTRAQ8plex (K) in Mascot. Peptide and protein was confirmed using scaffold (version Scaffold_4.2.1, Proteome Software Inc., Portland, OR). The False discovery rate (FDR) of protein identification was controlled at less than 5%. The DEPs (3 Vs 3) were identified with the criteria: fold change ≥ 1.2 or fold change ≤ 0.84, P < 0.05^26^,^27^. The functions of the DEPs were analyzed using bioinformatics approaches. First, Gene ontology (GO) enrichment analysis was performed using DAVID (https://david.ncifcrf.gov/)^28^. The pathways of the DEPs were identified using the Kyoto Encyclopedia of Genes and Genomes (KEGG) and REACTOME pathway databases. The key signaling pathways were illustrated using the Cytoscape software (http://www.cytoscape.org/)^29^.

### Western blotting

The 14 skeletal muscle samples were first lysed with cold T-PER Tissue Protein Extraction Reagent (Thermo, USA). Then, the protein concentration of the lysates was measured using the BCA protein kit (Beyotime, China). Thereafter, the proteins were sorted with SDS-polyacrylamide gel, and then transferred to polyvinylidene fluoride membranes (Millipore, USA). Then the membranes were blocked with 5% non-fat milk in Tris-buffered saline containing 0.1% Tween-20 (TBST) for 1 hour at room temperature. Subsequently, the membranes were incubated for overnight at 4 °C using primary antibodies against CYC1, NDUFC2, NDUFS3, PC, LDHB, GPI, PKM, TPI1, PAGM2 (Proteintech, China; 1:300 dilution), α-Tublin (Abcam, 1:1000 dilution) and GAPDH (Abcam, 1:1000 dilution). The HRP-labeled anti-rabbit/mouse IgG secondary antibody (Beyotime, China; 1:2000 dilution) was incubated for 1 hour at room temperature. Finally, the protein bands were visualized using the Immobilon Western Chemiluminescent HRP substrate kit (Millipore, USA). The gray values of the protein bands were measured using ImageQuantTL software (GE, USA). Levels of the α-tubulin protein were used as internal control. T-test was conducted to analyze the statistical significance of differences between low and high- FE pigs. Significance level was set at P < 0.05.

### Quantitative PCR (qPCR)

Total RNA of the 8 skeletal muscle samples was extracted using TRIzol reagent (Invitrogen, Carsbad, CA, USA). Reverse transcription was performed using M-MLV reverse transcriptase (Invitrogen). qPCR was carried out in a CFX96 Bio-Rad (Bio-Rad, USA) device using the SYBR Green PCR Master Mix kit (Toyobo, QPK201, Japan). Relative gene expression levels were calculated by 2^−ΔΔCt^ method. Levels of the α-tubulin mRNA were used as internal control. All of the qPCR primers used in this study are listed in Supplementary Table 3. T-test was conducted to analyze the statistical significance of differences between low and high- FE pigs. Significance level was set at P < 0.05.

## Additional Information

**How to cite this article:** Fu, L. *et al*. Proteomic analysis indicates that mitochondrial energy metabolism in skeletal muscle tissue is negatively correlated with feed efficiency in pigs. *Sci. Rep.* **7**, 45291; doi: 10.1038/srep45291 (2017).

**Publisher's note:** Springer Nature remains neutral with regard to jurisdictional claims in published maps and institutional affiliations.

## Supplementary Material

Supplementary Table 1

Supplementary Table 2

Supplementary Table 3

## Figures and Tables

**Table 1:** List of the top 10 up-regulated and 10 down-regulated DEPs between high-and low-FE pigs.

Accession	Full Name	Gene symbol	FC(H/L)	P
F1RP25	citrate lyase beta like	CLYBL	0.13	0.004
I3LK01	keratin, type II microfibrillar, component 7C-like	LOC100621639	0.33	0.01
F1SMS8	lectin, mannose binding 1	LMAN1	0.42	0.04
F1SGI7	keratin 75	KRT75	0.44	0.03
I3LDS3	keratin 10	KRT10	0.44	7.0E-09
L8B0V6	Immunoglobulin heavy chain	IGHG	0.45	0.0007
F1SGG3	keratin 1	KRT1	0.5	0.002
F1SGG6	keratin 5	KRT5	0.51	0.001
K7GNK7	mitogen-activated protein kinase 6	MAP2K6	0.57	0.03
F2Z521	platelet activating factor acetylhydrolase 1b regulatory subunit 1	PAFAH1B1	0.58	0.01
I3LLH4	SH3 and cysteine rich domain 3	STAC3	1.43	0.001
F1RUV5	pyruvate carboxylase	PC	1.44	0.02
F1RX51	malignant T-cell-amplified sequence	Ssc.3881	1.48	0.001
I3L8S3	carboxymethylenebutenolidase homolog	CMBL	1.5	0.01
I3LRS5	aldehyde dehydrogenase 1 family member A1	ALDH1A1	1.5	0.03
F1S288	syntrophin beta 1	SNTB1	1.57	0.04
Q2PYM7	proteasome subunit beta 9	LMP2	1.85	0.04
Q3ZD69	lamin A/C	LMNA	1.92	0.002
Q2QDF0	deoxyribonuclease I-like 1	DNASE1L1	2.14	0.01
I3LDT4	Uncharacterized protein	Uncharacterized protein	2.38	0.03
